# Recent Advances in Enabling Green Manufacture of Functional Nanomaterials: A Case Study of Bioinspired Silica

**DOI:** 10.1021/acssuschemeng.2c02204

**Published:** 2022-09-08

**Authors:** Robert Pilling, Siddharth V. Patwardhan

**Affiliations:** # Green Nanomaterials Research Group, Department of Chemical and Biological Engineering, The University of Sheffield, Mappin Street, Sheffield S1 3JD, United Kingdom

**Keywords:** System-based product design, Linked innovation, Formation pathways, Sustainability, Scale-up, Technoeconomics

## Abstract

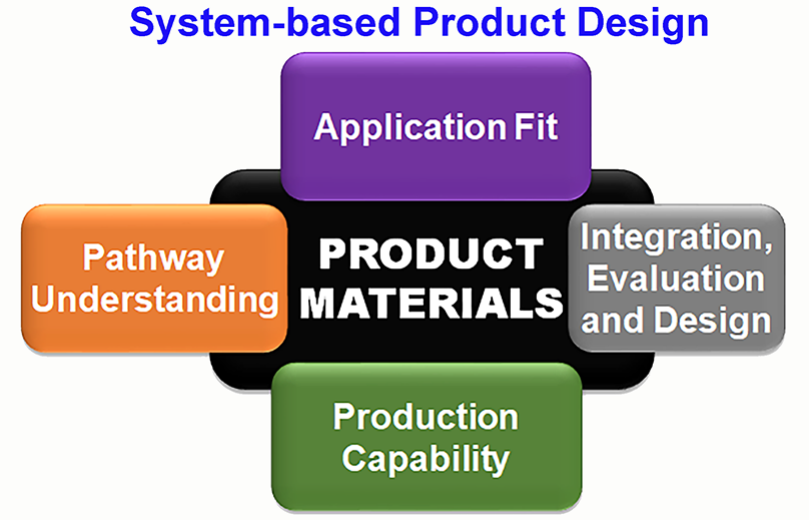

Global specialty silica production is over 3 million tonnes per annum with diverse applications across sectors and an increasing demand for more complex material structures and surface chemistries. Commercial manufacturing of high-value silica nanomaterials is energy and resource intensive. In order to meet market needs and mitigate environmental impacts, new synthesis methods for these porous materials are required. The development of the bioinspired silica (BIS) product system, which is the focus of this review, provides a potential solution to this challenge. BIS is a versatile and greener route with the prospect of good scalability, attractive process economics and well controlled product materials. The potential of the system lies not only in its provision of specific lead materials but also, as itself, a rich design-space for the flexible and potentially predictive design of diverse sustainable silica nanomaterials. Realizing the potential of this design space, requires an integrative mind-set, which enables parallel and responsive progression of multiple and dependent research strands, according to need, opportunities, and emergent knowledge. Specifically, this requires development of detailed understanding of (i) the pathways and extent of material diversity and control, (ii) the influences and mechanisms of scale-up, and (iii) performance, economic and environmental characteristics and sensitivities. Crucially, these need to be developed for the system overall, which sits in contrast to a more traditional research approach, which focuses initially on the discovery of specific material leads at the laboratory scale, leaving scale-up, commercialization, and, potentially, pathway understanding to be considered as distinctly separate concerns. The intention of this review is to present important recent advances made in the field of BIS. Specifically, advances made along three research themes will be discussed: (a) particle formation pathways, (b) product design, and (c) scale-up and manufacture. These advances include first quantitative investigation of synthesis-product relationships, first structured investigation of mixing effects, preparation of a broad range of functionalized and encapsulated silica materials and continued industrial engagement and market research. We identify future challenges and provide an important foundation for the development of new research avenues. These include the need to develop comprehensive and predictive product design models, to understand markets in terms of product cost, performance and environmental considerations, and to develop capabilities enabling rapid prototyping and scale-up of desired nanomaterials.

## Abstract 2

We demonstrate a novel integrative system-based product design approach for orchestrating multiple research strands and driving the realization of bioinspired silica nanomaterials.

## Introduction

### Specialty Silica

Global specialty silica production in 2015 was estimated at 3.3 million tonnes. In 2018, the market was valued at $5.2B, a figure which is projected to double by 2026.^1−3^ The main products, categorized by manufacturing route, are precipitated silica, fumed silica, microsilica, and sol–gel silica. These materials find diverse applications across different sectors, the largest being rubber, construction, agrochemicals, oral care, and food/feed. As markets evolve so do the requirements on the materials they depend on. With an increasingly prominent need for carbon capture and sequestration, advanced drug delivery systems, and next-generation catalysts and supports, more complex material structures and surface chemistries are required to meet the growing and specific demands. Commercial manufacturing of high-value nanomaterials is energy and resource intensive.^4^ In order to meet market needs and mitigate environmental impacts, new synthesis methods for porous materials are required and are the subject of significant scientific interest.

There are a number of methods for producing nanomaterials, which are classified broadly as top-down (destructive) and bottom-up (constructive) methods. The latter are becoming more popular as they confer advantages, including the ability to tailor composition, size, and structure. They also tend to produce less waste. Such methods are founded on the understanding and successful exploitation of mechanisms of nucleation and growth. Emerging methods such as templated self-assembly and combined “top-down/bottom-up” offer even greater potential both for controlling material properties and improving resource efficiency. The bioinspired silica (BIS) system described in this review represents an emerging bottom-up process in the form of a sol–gel process, adapted by the use of bioinspired additives.

### Bioinspired Silica Nanomaterials

**Figure 1:**
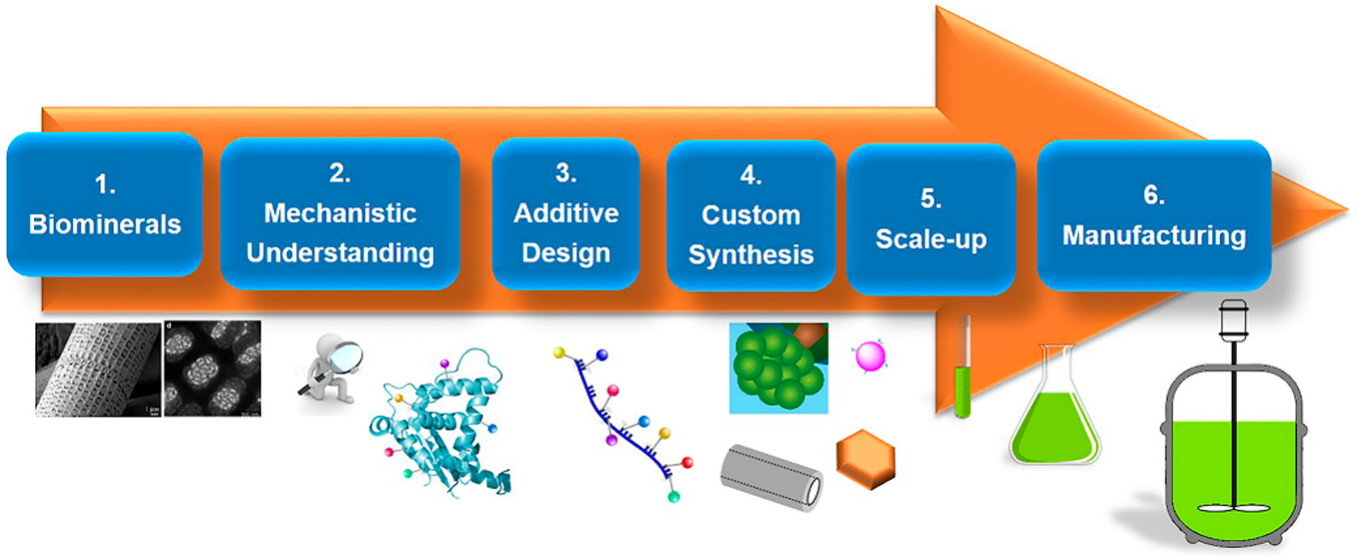
Bioinspired silica development pipeline. Image reproduced with permission from Patwardhan, S. V.; Staniland, S. S.^8^

Nature produces a wide range of nanomaterials under ambient conditions. Biological silica has interested scientists for centuries, due both to the intriguing structures of biosilica formed and to the corresponding formation processes. Diatoms are of particular interest, due to their hierarchical and varied structures. Biosilica is also found in Raiolaria, sponges, and many plants. These natural materials provide structural strength. Porosity may also be exploited to control transport of materials, or to encapsulate them. Research over the last two decades^5−7^ evolved from morphological observations to detailed elucidation of molecular and genetic mechanisms.^8^ Four key stages have been identified: (1) uptake of silica, (2) transport within the biological system, (3) biochemical transformation, and (4) deposition to the final location.^9^ Each stage was found to involve active processes mediated by specialized biomolecules. Once these had been identified, isolated, and purified, it became possible to investigate their role and function *in vitro*, in turn inviting studies on related synthetic materials, further expanding understanding of mechanisms and structure–activity relationships. The biological extracts and biomolecules themselves are not practical for industrial scale-up, but they have provided an inspiration for the design of simpler additives capable of mimicking the function of their more complex biological counterparts. Indeed, this bioinspiration led to the development of the bioinspired nanomaterials. With the example of the silica product system,^10^ this review reflects and builds upon the bioinspiration journey shown schematically in Figure 1.

A more detailed summary of the early stages of this pipeline for silica is provided by Patwardhan and Staniland,^8^ along with suggestions for further reading. Influential studies include those contributing to biological understanding,^5−7^ and development of synthetic systems.^11,12^ This review, recognizing both the progress that has been made and the opportunities now arising, develops and applies a complementary perspective, oriented toward commercial realization of what we have defined as the bioinspired silica (BIS) product system. The paper focuses on work and progress with regards to this reaction system (i.e., rather than its more complex biological counterparts).

### System-Based Product Design (SPD)

BIS may be recognized as a distinct product-system comprising materials derived from aqueous, low-temperature silica condensation, mediated by a range of bioinspired amine additives. As such, it represents a versatile and greener route with the prospect of good scalability, attractive process economics, and well controlled product materials. It thereby offers a potential solution to the material challenges described.^8^ Importantly, the potential of the system is recognized to lie not only in the properties of specific lead materials but also in its overall potential as a rich design-space for the flexible and potentially predictive design of a wide range of sustainable silica nanomaterials (the potential characteristics, scope and diversity of which are explored further by this review).

**Figure 2:**
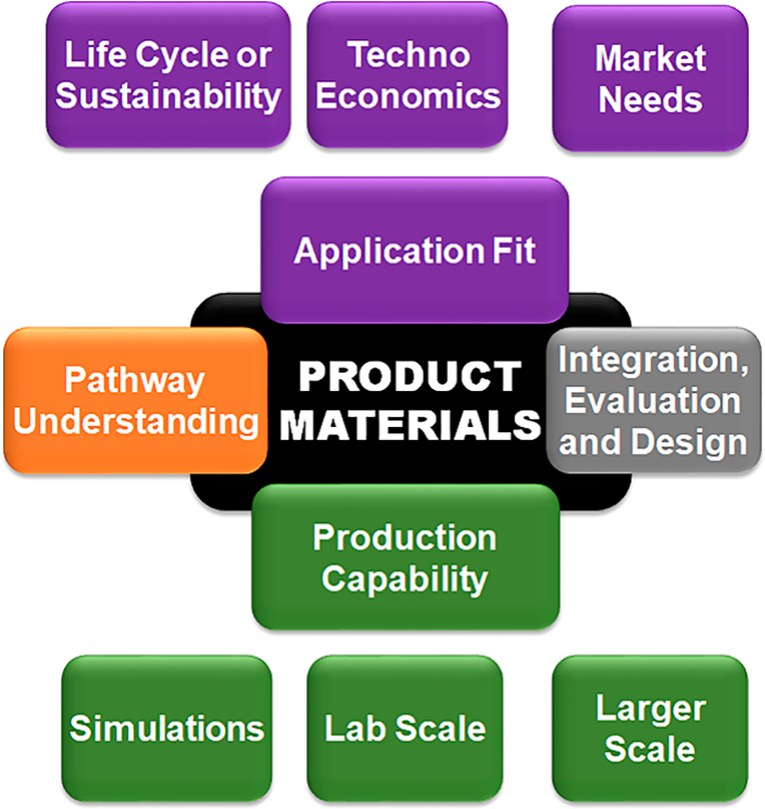
System-based product design research framework.

To realize this potential most effectively requires an integrative mind-set, which applies the principles of linked innovation,^13^ enables parallel and responsive progression of multiple and dependent research strands, according to need, opportunities, and emergent knowledge. Specifically, this requires concurrent development and understanding of (i) the pathways and extent of material diversity and control potential, (ii) the influences and mechanisms of scale-up, and (iii) performance, economic and environmental profiles and sensitivities. Crucially, these need to be developed for the system overall, which sits in contrast to a more traditional approach, which focuses initially on the discovery of specific material leads at the laboratory scale, leaving scale-up, commercialization, and, potentially, pathway understanding, to be considered as distinctly separate concerns. This viewpoint is neatly encapsulated by the principles of what has been termed system-based product design (SPD). Figure 2 lays out a corresponding depiction of our research framework.

### Scope of This Review

The review presents important recent advances made in the field of BIS and aligns these to our system framework (Figure 2). This illustrates the value of the latter, in the way it helps identify gaps, challenges, and future research for the BIS system. Also, by encapsulating the essentials of the associated research journey, it enables knowledge translation to other families of materials. Overall, we provide a systemic view of bioinspired silica and a structured view of recent research to investigate, develop, and exploit the system for the manufacture of silica nanomaterials. Both the framework and the featured research reflect specifically on achievements of the SynBio-Inspired Nanomaterials Manufacturing (SynBIM) project,^14^ and also on outcomes of the associated but wider Research Symposium.^15^

## Overview of Bioinspired Silica

In this section, we define and survey the BIS product system. First, by describing its broad scope in terms of synthesis, formation pathway, and material properties. Second by identifying and summarizing key design factors as product control, scalability, and performance, environmental profile and techno-economics. Finally, we consider associated research needs and direction. These aspects are then further described in subsequent sections with chemical and mechanistic details.

### Synthesis and Formation Pathway

**Figure 3:**
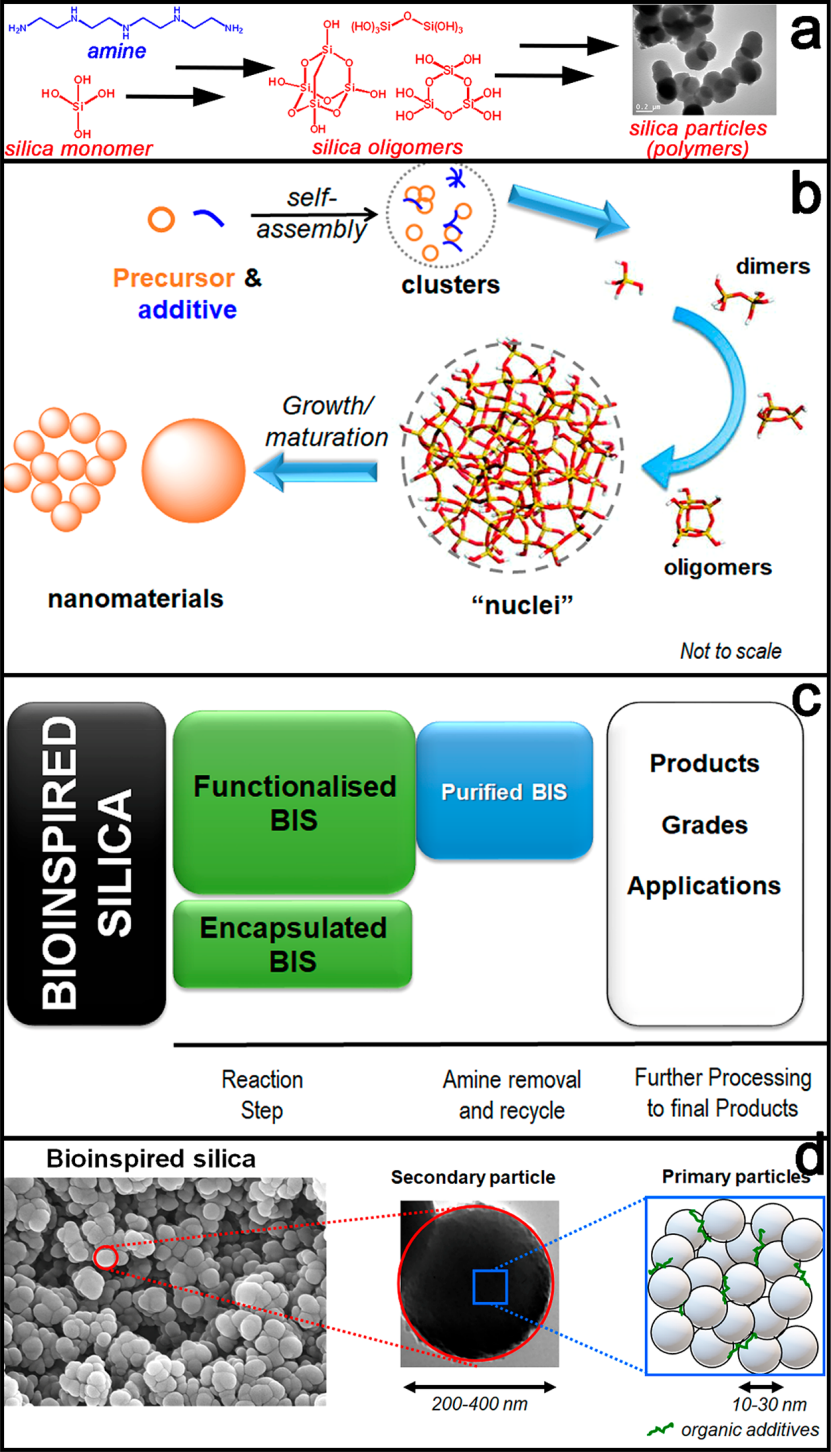
Overview of BIS synthesis, formation pathways, their compositional subtypes, and particle structures. (a) The chemical formation of bioinspired silica (image reproduced with permission from Dewulf et al.)^16^ and (b) the physical formation of bioinspired silica. (c) Three compositional subtypes of bioinspired silica. (d) Multiscale particle structure of bioinspired silica showing a representative SEM image (left) and TEM image (middle) of as-made BIS. Right schematic shows the primary particles and the additives aggregated. Image adapted with permission from Entwistle et al.^17^

The BIS product system is an example of a sol–gel process, which has been well studied and provides a versatile solution-based, low-temperature route to a range of silica nanomaterials. These bioinspired silicas are formed by condensation of silicate monomers, producing oligomers, which subsequently grow into solid polymeric silica particles that precipitate out of the reaction suspension. The reaction is mediated by a rage of amine additives, for example tetra-ethylene-pentamine (TEPA) as illustrated in Figure 3a. Attractive features of the approach are the mild conditions (aqueous, pH neutral, and ambient temperature) and, in comparison with the biological systems which inspired them, use of relatively simple additives. In combination, these offer the prospect of good scalability, attractive process economics (especially where the additives can be recycled) and well controlled product materials. These aspects are extensively reviewed elsewhere.^8,10^

### Formation Pathway

Silica precursors and the additive self-assemble to form clusters, which subsequently organize into nuclei, which in turn form product nanomaterials through growth and aggregation as shown in Figure 3b. The bioinspiration of BIS extends down to the molecular level, where mechanistic understanding of biomineralization provides the starting point for control, optimization, and design of the synthetic analogue systems. Natural systems employ a range of biomolecules to mediate formation processes.^18^ Although the details vary, a common feature of these systems is the presence of unique catalytic or binding sites, derived from specific amino acid sequences, which offer strong recognition (i.e., selectivity and high affinity). In turn, these mediate specific inter- and intramolecular dynamic self-assembly with inorganic species and enable controlled synthesis, assembly, and functionalization of nanomaterials.^19^ Over the years, synthetic additives, which mimic the function of such biomolecules, have been utilized.^20^ The simplified nature of the BIS additives compared to natural biomolecules reduces the potential complexity of mediating interactions; however, their success in mediating formation processes demonstrates that, nonetheless, they play an active role. Recent structure–activity investigations (detailed in later sections) suggest that a key feature, which enables BIS additives to facilitate silica formation under ambient conditions and neutral pH, is their ready ability to form water-soluble cations via dynamic and reversible protonation. Beyond this, the structure, architecture, amine environment, and additive length all play a role in controlling additive–silicate self-assembly and catalysis of silicate condensation/particle formation and materials properties.^8,21^

### Material Properties

As BIS forms from monomers building into complex polymeric particles (Figure 3c), subsequent removal of water provides dry materials, generally taking the form of free-flowing white powders. After the main reaction step, the amine additives remain incorporated within and on the surface of the nanomaterials, thereby offering a single step route to functionalized silica (shown in Figure 3c). With modification to the process (i.e., including metal catalysts, biologicals, drug molecules, etc. in situ), encapsulated silicas can also be produced.^22^ Alternatively, the additives may be removed via the new discovery of a mild room temperature acidification to provide pure silica products with associated recovery of the valuable additives for reuse.^23^ These options offer three compositional subtypes of BIS final materials (Figure 3c)

BIS materials exhibit intrinsic structure and morphology over a range of length scales, in large part, these features are characterized in terms of particle size/shape and porosity (care is needed with terminology, however, in order to accommodate the complexity created by multiscale and cross-disciplined characteristics). Higher order structures are also possible, though have not, as yet, provided a central focus for research. BIS materials may thus be usefully characterized in terms of composition, particle properties, porosity, and higher structure. These characteristics are further summarized below.

The term “particle” is applied to define the smallest structural unit for a given length scale. Particles corresponding to respectively increasing length scales are differentiated by the qualifiers: primary, secondary, and tertiary. Primary BIS particles tend to form in the 5–10 nm range, although up to ∼25 nm particles have been observed (see Figure 3d).^17,24^ These represent the basic physical building blocks of the corresponding macro-scale white powders. Secondary particles form through aggregation (i.e., with associated chemical interlinking of primary particles) as visualized using electron microscopy, falling into the 200–400 nm size range. Tertiary particles form at larger scales and simply reflect the agglomeration (i.e., no chemical interlinking) of secondary particles through the precipitation and drying steps.

**Figure 4:**
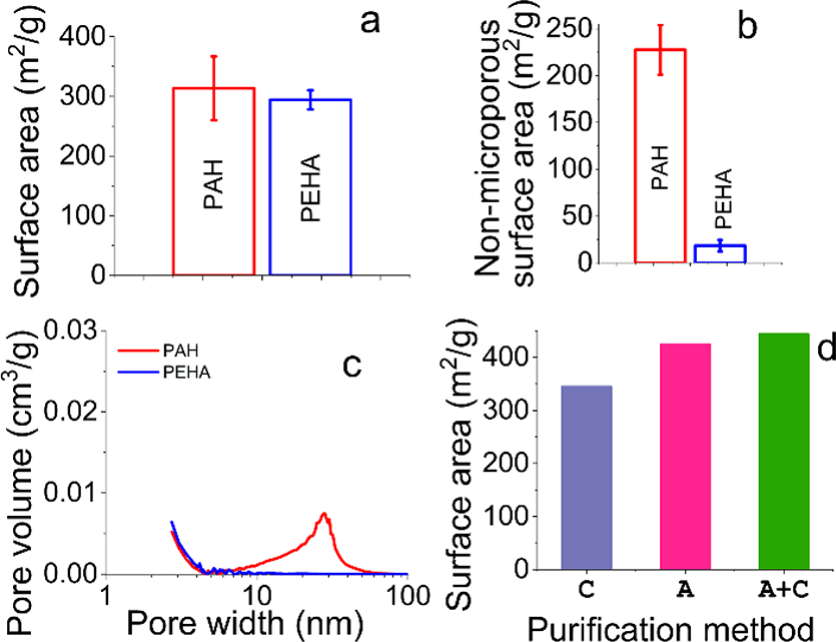
(a–d) Porosity of bioinspired silica when using different additives (a–c). (d) Removal of the additives/purification via Calcination (**C**), acid elution (**A**) or acid elution followed by calcination (**A+C**). Images reproduced with permission from Routoula (a–c) and Manning et al. (d).^23,25^ Note that the difference between samples denoted **C** and **A** (or **A+C**) in Figure 4d is from the method, not the extent of removal (which is ∼100% for both). Calcination is known to create local “explosions” with the additives burning and leading to fracturing some pore walls/damaging pores, hence higher specific surface area.

Porosity refers to the size, shape, distribution, and order of void space within physical materials. Pores are categorized by pore diameter: micro (<2 nm), meso (2–50 nm), and macro (>50 nm). Associated size distributions are described by measures of polydispersity. Porosity may also be considered in terms of its influence over bulk properties such as surface area and pore volume. When using small amine additives (e.g., pentaethylenehexamine, PEHA), standard functionalized BIS products (i.e., still containing the additive) are formed, which are categorized as low surface area microporous materials (i.e., porosity may extend into even mesoscale but the microscale dominates: blue data in Figure 4a,b,c). Removal of the additive provides a further increase in microporosity (Figure 4d). When larger polymeric additives such as polyethylenimine (PEI) or poly(allylamine hydrochloride) (PAH) are used, porosity in the mesoscale is generally obtained with higher surface area, before and after the removal of the additives (red data in Figure 4a,b,c). Various silica materials are known, which exhibit porosity in the mesoscale. However, the term “mesoporous silica” is generally understood to refer to materials that exhibit porosity predominantly in the mesoscale and with monodispersity. The common standard for this being MCM-41. It is likely that future work leads to the preparation of such a mesoporous BIS (i.e., predominantly mesoscale pores with tight dispersity).

Beyond intrinsic particle properties and porosity, silica nanomaterials may also exhibit a higher order structure. Although, BIS development has centered on the preparation of freely formed particles and gels. Modifications have been demonstrated using prebound additives for the preparation of coatings. More advanced bioinspired control strategies have the potential to offer further structural complexity and selected examples are detailed in ref (11).

**Figure 5:**
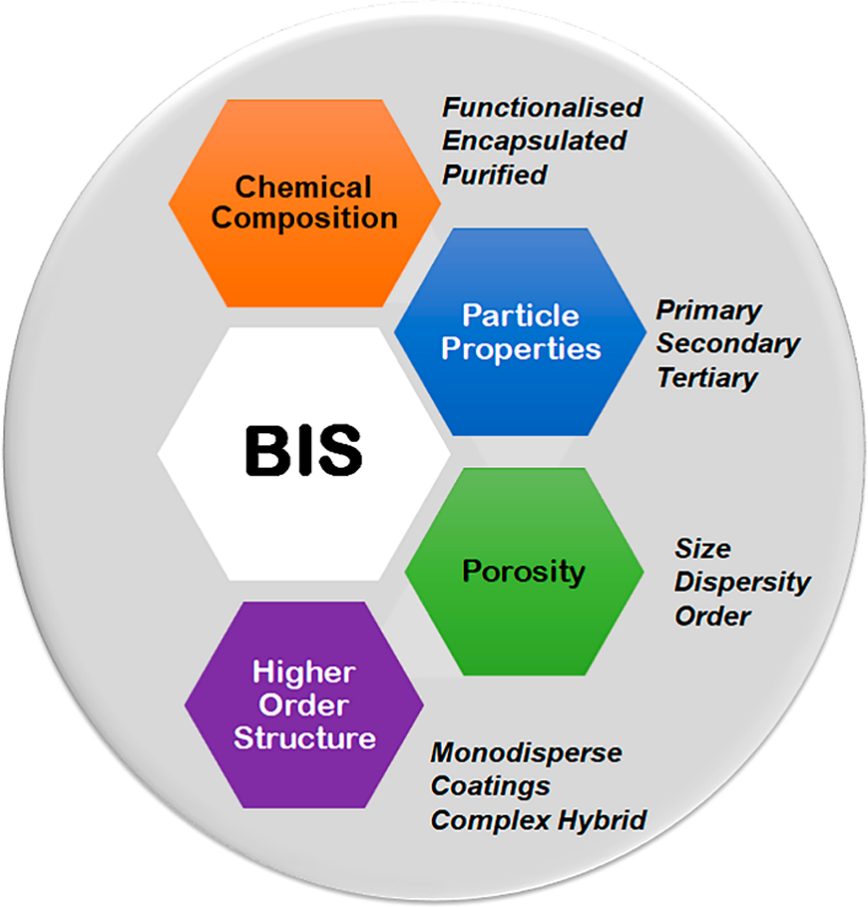
Selected characteristics mapped to a broad depiction of the BIS material design space.

The preceding summary serves to illustrate the versatility of the BIS system in its ability to offer diverse products and material from a single platform technology. Conceptually, this “product space” may also be considered as a “material design space” for targeted applications (Figure 5).

### Product Control

**Figure 6:**
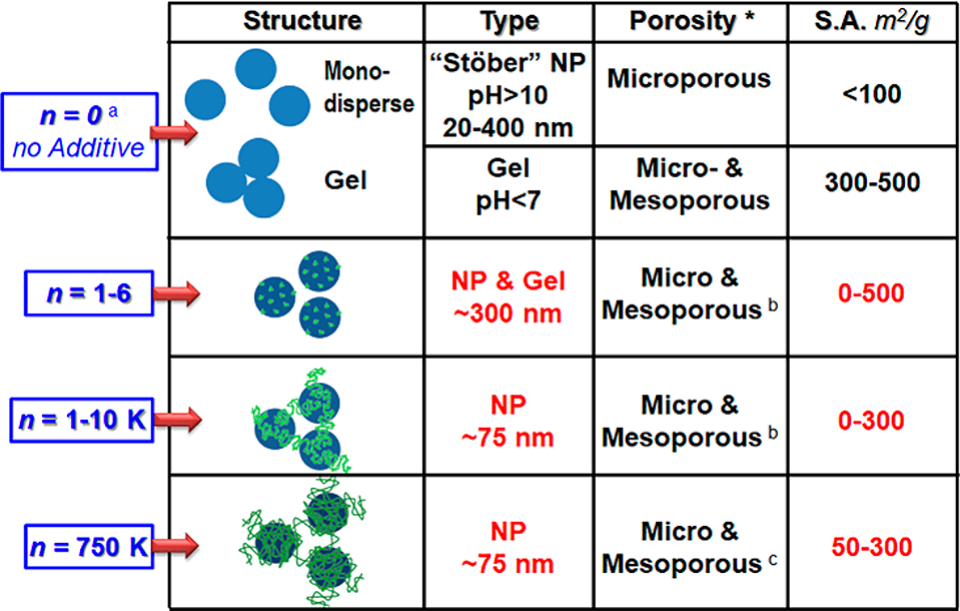
Schematic showing control of the properties of biosinspired silica with an example of ethyleneamine additives. Properties in red colored text can be tuned by varying parameters in blue text. ^a^*n* denotes the number of repeat units in the ethyleneamine additives used. ^b^Room temperature acid elution removes additives (fully for smaller and partially for larger additives, for details, see refs (23 and 26)). ^c^Calcination is used for fully removing larger additives. *Refers to internal porosity and excludes external porosity arising from interparticle pores.

Building on the idea of a BIS design space, Figure 6 illustrates aspects of the bioinspired control that allows us to navigate it, with an example of one family of additives where the additive size can be used to tune BIS porosity and composition/functionality.

### Process and Scale

The formation of additive-containing BIS materials occurs in one step, and a second mild acid elution step has been developed to proceed to the pure form with an additive recycle.^23,26^ After these two novel steps, filtration and drying, along with further downstream processing options converge with standard and well-established methods for materials of this type, hence enabling retrofitting and avoidance of additional capital costs. Through a combination of experiments and simulations, the acid mediated removal of additives was found to be dependent on additive-surface binding and additive solvation.^23^

**Figure 7:**
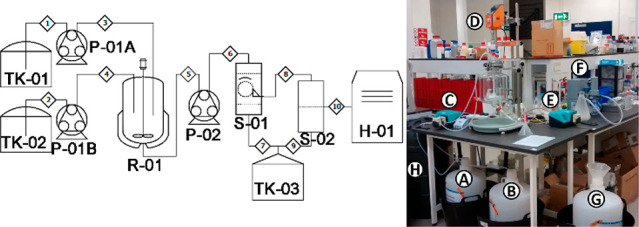
(Left) Process flow diagram and (right) photo of larger scale BIS production apparatus operated in a 5 L continuous stirred tank reactor. The units are marked as follows. A, B - feedstock tanks (TK-01, TK-02); C, E - pumps (P-01, P-02); D - reactor (R-01); F - filtration (S-01, S-02); G - waste filtrate tank (TK-03); H - overflow tank; H-01 - drying oven. Images reproduced with permission from Manning.^27^

BIS synthesis has been demonstrated successfully from the 2 mL to the 40 L scale in batch stirred tanks (up to 1 kg product).^22^ Consistent recoverable yields were obtained across this range, along with relatively consistent product properties. The process is also adaptable for continuous manufacturing and it has been demonstrated using continuous stirred tank reactors (CSTRs) and plug flow reactors (PFRs) (Figure 7).^27^ These results provide a good confidence of scalability across the BIS product family and also for further scale-up using desired configurations toward full pilot and industrial regimes. As such, these results also represent a successful journey along the pathway shown in Figure 1.

### Performance, Economics, and Environment

While the headline figures of production scales and market values noted earlier for specialty silica illustrate incredible scale, understanding the potential for bioinspired silica nanomaterials to compete within, and indeed to influence the makeup of this market, requires a more detailed view. Establishing and interrogating this is its own research challenge. Thus, the viability of a specific product within a specific application-market turns on its corresponding specific performance, economic, and environmental characteristics.

In general terms, however, BIS material performance depends most strongly on porosity (surface area, pore size, and pore volume) and to some extent on the particle nature (size, sphericity, and dispersity). Beyond this, the specific morphology is less critical. For some applications, such as cosmetics and tires, pH (determined by levels of surface silanols and additives) can influence product compatibility. Purity and yield are universally important. The former, particularly because additives and associated salt byproducts are occluded in the final silica products. The latter due to the often poor resource efficiency and high waste production of many published methods, and consequent implications for economic viability and environmental profile. From a manufacturing standpoint it is important that materials can be produced at scale. Traditionally scale-up has been considered as an afterthought to lab-scale discovery and product evaluation. Increasingly, it is recognized to be important and efficient to consider scale-up viability and process sensitivities from the outset. Thinking even more systematically, and from a commercial perspective, materials must be scalable, economically viable and offer a good environmental profile. Economic and sustainability analyses provide corresponding analytical frames and our work has highlighted the importance of their incorporation at the discovery and design stages.

With regards to understanding the environmental component of this picture, the principles of green chemistry provide an umbrella of opportunities for driving improvements. While detailed life-cycle analysis offers a robust, if labor intensive, means to probe implicated value, sensitivities and potential trade-offs. The devil as so often is in the detail. An important challenge for pursing a more integrated and systematic design and development approach from the outset lies in balancing the added complexity, with the need to make practical progress. There are now a raft of specific green chemistry metrics,^4^ which may be deployed to support these efforts, though we do not dwell on their detail here.

For the purposes of this review, we have concentrated on the comparison of BIS materials with corresponding commercial or laboratory counterparts. With respect to the consideration of the supply of the silica precursors, they are relatively common between these different routes and hence their inclusion into the comparison of different types of silicas is not included. We have also defined the BIS product system as described, which reflects that, based upon current understanding of formation pathways, an effective “nonamine-based BIS system” is unlikely in the extreme.^28^

We have emphasized energy and material efficiency in our discussion since this is where most comparative information is currently available. There is also now a recently published study,^4^ which quantifies the environmental impact for each of the 12 principles of green chemistry. This appears to be a useful tool for both broadening and integrating early stage thinking on environmental impacts. This preliminary work suggests that with regards to the overall sustainability score, there is a relatively low sensitivity to the use of amine additives in BIS and other silicas. Any differences between the amines used for the different types of silicas were found to be outweighed in the overall score by the design for energy efficiency rating criteria, in which BIS does particularly well.

Techno-economic analysis, meanwhile, indicates that the BIS synthesis can reduce energy use of the reaction step by 95% compared to industrial precipitated silica.^23,29,30^ Also, that the cost would be comparable to the lowest grade of this commercial counterpart, while providing significantly better product quality and properties. The more recently reviewed mild purification step is crucial, since the alternative calcination route is energy intensive. For example, respective approximate CO_2_e/ton SiO_2_ for manufacture of bioinspired silica are estimated at 0.4 (calcination), 0.9 (ethanol reflux), and 0.01 (mild acid). The mild purification route provides a 97% reduction of energy over calcination. Process advantages and economic benefits are even more impressive in comparison with current methods for accessing higher valued products, such as mesoporous drug delivery agents. These are highlighted in Table 1, while further details can be found elsewhere.^10^

**Table 1:** Comparison of BIS with MCM-41 as a Current Benchmark Material, Adapted from Ref (10)

			Reaction conditions							
Silica type	Reagents^a^	Solvents	*t*	*T* (°C)	pH	Byproducts	*E*-factor^b^	Yield^b^ (g L^–1^)	Finishing	Cost (£/kg)
Mesoporous - MCM-41^31^	Silicate, CTAB, ammonia	Water	2–6days	20–120	∼9	Alcohol, NO_*x*_, CO_2_	12 (60, 900)	17	Calcination (550 °C, 6 h)	>1000
Bioinspired	Silicate and additive	Water	5–20min	20	7	NaCl	5 (30, 85)	Up to 38	Centrifuge, dry	1.5–5

a Silicate = sodium silicate or water glass, CTAB = cetyltrimethylammonium bromide.

b Calculated from representative internal results; numbers in parentheses include reaction solvent, reaction solvent + washing/purification solvent.

### System Research Drivers

This section provides an overview of bioinspired silica by introducing the synthesis method, formation pathways, and structure and properties of BIS materials, and also associated design considerations. Conceptualising this as a product system allows the consideration of the corresponding overall set of possibilities from related but distinct perspectives. Specifically, it is possible to understand and work with the system level concepts of reaction space and product (design) space. It also enables the consideration of the extent to which we are able to describe, explain, and predict how these two spaces relate in terms of pathway understanding. Finally, the practical dynamics can be considered of research, development, evaluation and design, and the ways in which knowledge is conceptualized, captured, managed and progressed.

This system model does not provide a traditional or mechanical framework, for how things happen, rather it lays out possibilities, space, and potential relations and dynamics. It is thus more useful for organizing and exploring thinking, than defining concrete processes or blue prints. Thus, it offers a systematic and structured yet also fluid and responsive research frame. One implication is that it may be helpful to consider research needs or drivers in terms of the corresponding components: production capability, pathway understanding, application fit, and integration, evaluation and design. One feature of this typology is that it is product-centric rather than “discipline-based” or “challenge-driven”. One might consider this as a “middle-out” formulation rather than the latter’s respectively bottom-up or top-down. As such, it offers a complementary rather than competing view. Therefore, we can see how our system-based research drivers align with the key challenges identified for realizing the potential of BIS materials through the concurrent development of a fundamental understanding of (i) the pathways and extent of material diversity and control, (ii) the influences and mechanisms of scale-up, and (iii) the performance, economic and environmental characteristics of the system’s overall product potential. As we will see, significant progress has been made toward these goals. Important recent advances are presented, while also commenting on associated knowledge gaps and opportunities. These advances and future challenges are then drawn together and summarized, by way of conclusion.

## Production Capability

### Reaction Space

The BIS system offers a rich parameter space for the exploration, optimization, prediction, and ultimately rational design of green bioinspired structured silica nanomaterials. This space may be considered in terms of control factors and product characteristics. Chemical factors comprise choice of additive, silicon precursor, reagent concentrations and ratios, and pH. Physical factors are reaction time, temperature, addition order/rate, mixing type/rate, reactor type, and geometry and configuration. Tables 2 and 3 describe the relevant reaction space considered in terms of chemistry and physics.

**Table 2:** Chemical Reaction Space for Bioinspired Silica Preparation

Chemistry	Relevant range/options	Notes
Amine additive	Varying origins (natural, biological, synthetic), molecular sizes (small, polymeric), architectures (linear, branched), and chemistries (N substitution, modifications).	There is a huge range of additives used, and additive “type” has a profound influence on silica formed.
Silicon precursor	Alkoxysilane, sodium silicates, Si-catecholate complex, modified silanes	Need to consider what trigger is required to start the reaction, effects on reactions/products, scalability, sustainability, and costs.
Ratio of precursor to additive	[Si]:[N] = 0.5–16	Main focus has been on the ratio of 1, but some studies used <0.5.
Reagent concentration	[Si] = 20–100 mM	There are also studies at <20 (8 and even 1 mM) and >100 mM (up to 660 mM).
pH	6–8, some at as low as 4	Generally controlled with a buffer (if not using sodium silicate) or simply by neutralization.
Others	Use of cosolvents and the type of acids used for hydrolysis	

**Table 3:** Physical Reaction Space for Bioinspired Silica Preparation

Physics	Relevant range/options	Notes
Reaction time	Typically 5–60 min	Also <1 min and >1 h (to a day or more)
Temperature	∼20 °C	This is generally kept constant but can be easily varied
Addition order/rate	Rarely studied	We found the order makes a difference (e.g., acid added to Si-source or amine or after mixing Si and N).^32^
Mixing type/rate	Rarely studied/completely unknown	Preliminary work shows that flow (*Re*) and mixing energy dissipated (ε) will affect the reaction and products.^33^
Reactor type	Batch, micro- and milli-fluidic devices, plug flow reactor	Very little understanding available
Others	Separation and drying also has an impact on the properties of silica produced.	

### Research Focus

Our systems-based product design approach integrates low and higher technology readiness levels (TRLs). At low TRLs (e.g., 1–3), there are two main drivers for exploration of a system’s reaction space. First is the desire to discover or design products with useful or valuable properties and second is the need for evidence to aid elaboration of physicochemical mechanisms (at different length- and time-scales spanning self-assembly to the final powder). For higher TRLs (4–7), the emphasis shifts toward multicriteria optimization of properties, performance, yield, economics, and environmental profile, enabling targeted product and process development and driving commercial uptake.

For the BIS system, prior knowledge of the reaction space derived largely from discovery work, which tended to pay little attention to purity and yield, and also to overemphasize the characterization and control of morphology (albeit such work does offer insight into associated growth mechanisms).^11^ Overall, the knowledge provided was diffuse, qualitative and addressed only one factor at a time. While this offers general insight, it is inefficient, overlooks second (or higher) order interactions, and fails to provide an effective strategy toward fully understanding or exploiting the system. For example, combined requirements for yield and surface area present a classic multidimensional optimization challenge, which is further complicated by taking scalability, process design, economics, and environmental impact into account.

By contrast, quantitative and systematic experimentation offers a path to deeper holistic understanding and optimization, including for interaction between multiple factors. In pursuing systematic approaches, it is important to maintain clear sight of the underlying research questions and to track advances against them. For the BIS system, these research challenges reflect the critical characteristics and considerations identified earlier and may be summarized as (1) elaboration of a BIS product-by-design space emphasizing porosity, purity and yield, (2) understanding associated scale-up characteristics and implications for process design, and (3) understanding and refining this overall design space from the perspectives of economics and sustainability.

### Synthesis–Product Relationships

Properties of bioinspired silica are found to depend on multiple synthesis parameters noted in Table 2. For some systems, silica yields are found to increase with reaction time (e.g., 100 mol % after 5 min) and also with increasing amine concentration (i.e., decreasing Si:N ratio).^16,34,35^ While for others, a drop from pH 7 to 6.65 decreased the yield substantially (from 66 to 47 mol %). Parameters such as the pH and [Si] have generally been kept constant within and between different studies. Both small straight chain (e.g., tetraethylenepentamine - TEPA) and polymeric (e.g., polyethylenimine - PEI) amines have been found to produce yields of around 50 mol % and a range of surface areas from 10 to 700 m^2^/g.^21^ Larger surface areas are potentially associated with shorter mixing times, decreasing additive length, and increasing Si:N ratios.

**Figure 8:**
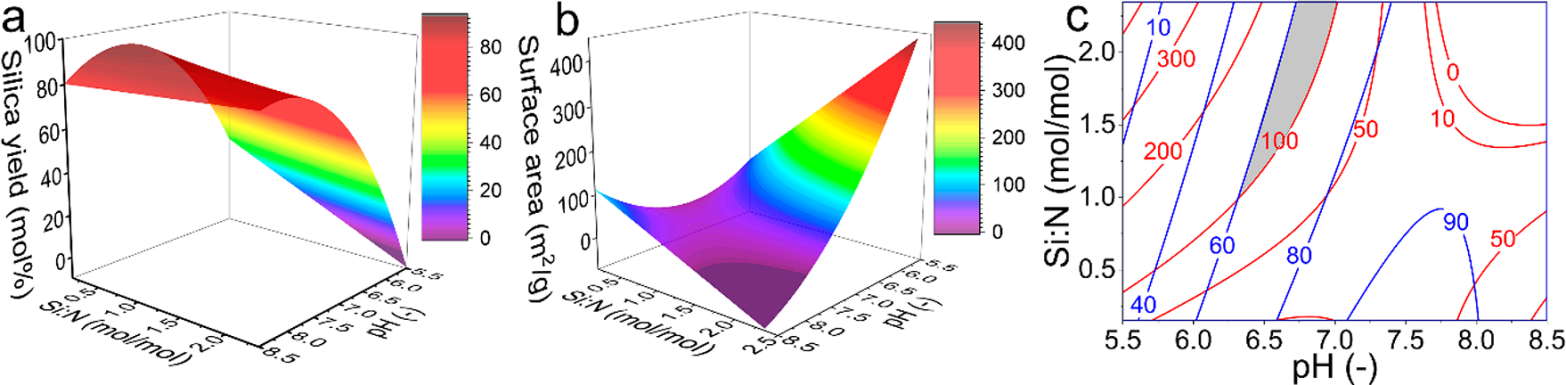
Three-dimensional response surfaces for (a) the silica yield and (b) the Brunauer, Emmett, and Teller (BET) surface area. (c) Overlaid contour plot of the model for silica yield (blue) and silica BET surface area (red) for optimization of both responses simultaneously. The gray region enables to synthesize silica with the constraints that the yield should exceed 60 mol % and the BET surface area should exceed 100 m^2^/g. Figures reproduced with permission from Dewulf et al.^16^

Recent research has made the first steps in a more quantitative and systematic direction, providing new insight into BIS reactions and products along with important methodological developments with potential for building upon in future work. One avenue explored was the development of a new sequential DoE strategy that included prescreening experiments, a successive screening using full factorial design and optimization.^16^ Out of the initially investigated three factors (Si:N, pH, and [Si]), in the range explored, only the first two were found statistically significant for silica yield and surface area. For complementation, a variance-based global sensitivity analysis using machine learning was successfully applied to bioinspired silica for the first time, which rapidly identified key parameters and interactions that control the physicochemical properties and corroborated DoE outcomes. While a maximum yield of 90 mol % and a maximum surface area of 400 m^2^/g were independently reached, this study clearly highlighted the difficulty in simultaneously achieving such high yield and surface area. Since for successful commercialization, high yields and large specific surface areas are desirable, their simultaneous optimization when focusing on only one additive, led to BIS with yields little over 60 mol % and with surface area just above 100 m^2^/g (see Figure 8).

The combined use of DoE and machine learning for inorganic materials synthesis is a research frontier, aiming to tackle the complexity inherent to materials design. Future research on BIS using this strategy will benefit from extending the parameter range (e.g., higher [Si], wider pH range) and including additional factors such as different additives and mixing. Further quantitative understanding of the nonlinear trends in factors, e.g., pH and Si:N as identified by statistical methods, is important for building a comprehensive optimization framework. This strategy is also transferable beyond this work.

### Scaling Up

**Figure 9:**
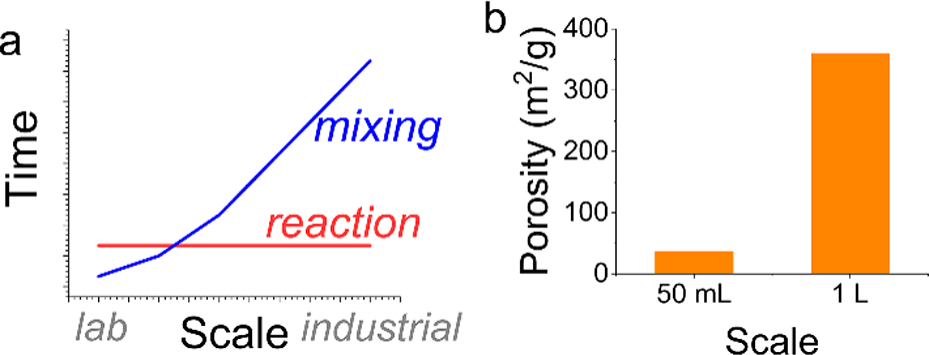
(a) Schematic representation of mixing and reaction kinetics change with scales. (b) Sensitivity of bioinspired silica products to scale-up.

Mixing effects are an important known cause of product sensitivity to scale-up. Simplistically, while reaction rates are independent of scale, mixing rates are not as shown schematically in Figure 9a. Consequently, critical balance between the two (defined as Damköhler number, *Da* = reaction time ÷ mixing time) is influenced by scale, thereby affecting resultant product characteristics. The larger scale synthesis referred to earlier, was attempted using a “traditional” approach (i.e., informed trial and error). The work successfully demonstrated general viability of scale-up but did so by employing crude intensive mixing. From a techno-economic perspective, mixing is potentially the largest energy input to the reaction step. Therefore, finer calibration and control is necessary for optimizing processes at scale.

Indeed, excessive mixing is also known to limit particle aggregation, leading to reduced particle sizes and an associated tendency to remain in suspension.^36^ Also, in earlier trials, surface area was found to change significantly with scale, yet unpredictably (Figure 9b). While an increase in surface area may be desirable in terms of performance, the unexplained variability introduces corresponding uncertainty into the translation of processes from small to large scales.

Building upon these initial findings, a structured and scientific approach needs to be adopted, seeking to understand the underlying influence of mixing effects over synthesis–product relationships. The ultimate aim being to develop predictive design capabilities, transferable across different process scales and also different product types and grades (e.g., as attention turns from one generic model system to more precisely defined product grades, it is important that, once predicted or established accurately at small scale, this precision can be translated reliably to larger scale). A good start to investigate the effects of mixing on the bioinspired silica process and products is by varying stirring conditions and feed points using an established methodology.^37^ Further analysis can provide both spatial and time-resolved mixing data.^38^ An in-house protocol for analyzing degree of mixing applied to the results indicates that bioinspired silica formation is controlled significantly by mesomixing, and to some extent by micromixing.^33^ The point of control was identified as the process of conversion from oligomer to particles (see Figure 3b). In contrast, both the conversion of monomers to oligomers and also to the final product were found to be insensitive to mixing conditions.

Most processes reviewed are governed by micromixing. Detection of the influence of mesomixing for the BIS system provides valuable insights into the underlying process with implications for scale-up.^37,39,40^ Identifying the point of influence as the conversion of oligomers to primary/secondary particles indicates that this step occurs on time scales similar to mixing timescales. Consequently, the associated nucleation/aggregation processes are sensitive to this regime. Conversely, it is concluded that the consumption of monomers is not.

Important details of these mesoscale dependencies are yet to be established, including crucially, the Damköhler number (i.e., ratio of mixing time to reaction time). This limits calculation and modeling capabilities such that it is not possible to predict the accurate scale-up behavior and product properties. Advances from mixing analysis as stated provide the foundation for future work that aims to establish the correlation of mixing mechanisms, chemical kinetics, and product properties using a combination of experimental data and computational fluid dynamics.^41^ The advances reported have also served to create a test bed for rapid process development and scale-up for a wider range of nanomaterial, which is crucial for speeding-up commercialization of various materials. Specifically, BIS synthesis at the 40 L scale was demonstrated and capabilities were created to routinely producing up to 100 g quantities for testing for other applications. Wider potential and impact of these capabilities is demonstrated by recent work on metal organic nanosheets (MONs), a completely different type of material, which has resulted in rapid demonstration of MON scale-up and a greener process was designed at low temperature, yielding high amounts of high-quality MON.^42^

## Pathway Understanding

### Mechanistic Understanding

Green synthesis of nanomaterials has been of interest for some time. Until recently, systems have been limited to simple reaction schemes where the additives (e.g., citric acid or tea or plant extracts) act as a reducing agent in the synthesis of metal nanoparticles (e.g., gold and silver).^43^ However, for most practical or commercial applications, materials of interest derive from more complex reactions. Bioinspired approaches for the synthesis of nanomaterials have been introduced as an alternative, however their mechanisms were rarely investigated owing to the complexities associated with the interactions between additives and precursors/intermediates/particles, which lead to unusual or non-traditional reaction pathways.^44^ In earlier work, an unproven multistep nonclassical formation pathway was postulated (see Figure 3b). In terms of understanding the BIS reaction pathways and, more specifically, elucidating the molecular-scale mechanisms that underpin synthesis–property relationships, two avenues of inquiry have been pursued recently:

(1) a sequential DoE strategy for systematic and quantitative investigation of the BIS system’s reaction space and^16^
(2) a combination of experimental measurements (advanced characterization, including solid state NMR^45^) and computation (multiscale modeling, including molecular dynamics simulations^46^) to investigate the additive-silica interface at molecular length-scales.

The DoE investigation, previously summarized, crucially demonstrated and quantified the pH dependency of the system. These observations align with, and are explained by, the findings of the second strand of investigations, which determined that the formation of mesoscale structures in solution are defined by charge-matching of ionic interactions between additives and silicates,^46^ and that the factors controlling these interactions are the additive concentration, pH, and reaction mixture ionic strength. These findings, which prove the multistep nonclassical formation pathway,^47^ have profound implications for the synthesis of a wide range of templated porous materials, not least, the observed pH sensitivity of the interface. Specifically, this implies that

- It may be possible to synthesize a family of porous silicates and nonsilicates with increasing degree of order by slightly altering the pH, thereby tuning the additive self-assembly.^28^ As controlling porosity is the holy grail for silicas, such products have particular and important potential for medical and environmental applications.
- The removal of additives or templates from product materials can also be tuned in a similar way. Indeed, the postsynthesis extraction of additives has been successfully tuned by simply changing the solution pH.^23^ The mechanistic insight also implies that this approach can be extended to tailor and predict purification of templated materials beyond silica as recently reported by Bedford and co-workers.^48^

In a further study, advanced characterization (ssNMR) of BIS identified unexpected, yet significant association between the additives and fully condensed (uncharged) surface silicon species, leading to higher levels of condensation and greater hydrothermal stability than benchmark materials (despite BIS products having a shorter synthesis time).^45^ These effects are attributed to the polyfunctional and catalytic nature of the additives. The charge-matching initiates additive-silica interactions at multiple surface sites followed by corresponding catalytic condensation of SiO^–^ moieties at multiple sites. These supplementary interactions mean that, mechanistically, the nature of the interactions are more complex than was previously anticipated. These results emphasize that BIS lies at the intersection between biosilica and artificially templated silica materials. BIS provides the advantages of a catalytically active templating behavior to artificially templated silica materials while enabling systematic investigation into the more complex interfacial phenomena present in biosilicas. These findings are significant as they demonstrate an as-yet unexploited design space for artificially templated silica materials, provide a potential explanation for the biological phenomenon of biosintering in sponge spicules, and highlight overlooked details in current atomistic simulations of templated silica interfaces.

Taking the available mechanistic evidence into account, it becomes clear that BIS additives self-assemble or coassemble with silicates, thereby catalyzing and templating final product structures.^20^ Also that different additives interact differently and perhaps selectively with the distinct stages of silica formation.^49^ Consequently, multiple modes of action are possible, including catalysis and stabilization, each with independent influence over product properties, as hypothesized over a decade ago.^20^ This conceptualization has stood the test of time with new evidence emerging. It is now known that this catalysis is enabled by dynamic protonation–deprotonation, which in turn influences aggregation so as to enable a form of self-assembly, and subsequent templating (or scaffolding). The recent research summarized has helped understand which additives perform catalysis and, in broad chemical terms, how this catalysis proceeds. Further, this research has identified the need to quantitatively measure the chemical kinetics (detailed in the following).

It is illuminating to track this evolution from postulated pathways and modes of action, through increasingly supported theories and toward physically defined and quantitatively determined mechanisms. Also, to recognize that this represents over ten years of intensive research. Through this period neither the driving questions nor their broad answers have changed dramatically, yet associated insight, knowledge, and practical capabilities have risen incrementally throughout. The ultimate goal remains that of establishing a level of detailed mechanistic understanding that enables predictive design, control and scalability of BIS materials and their properties. This challenge has become increasingly well-defined and understood, specifically, defining what is known and what is not, how and by what methods these gaps can be addressed, and how and to what ends a complete and quantitative picture may be established.

### Unified Mechanistic Understanding

**Figure 10:**
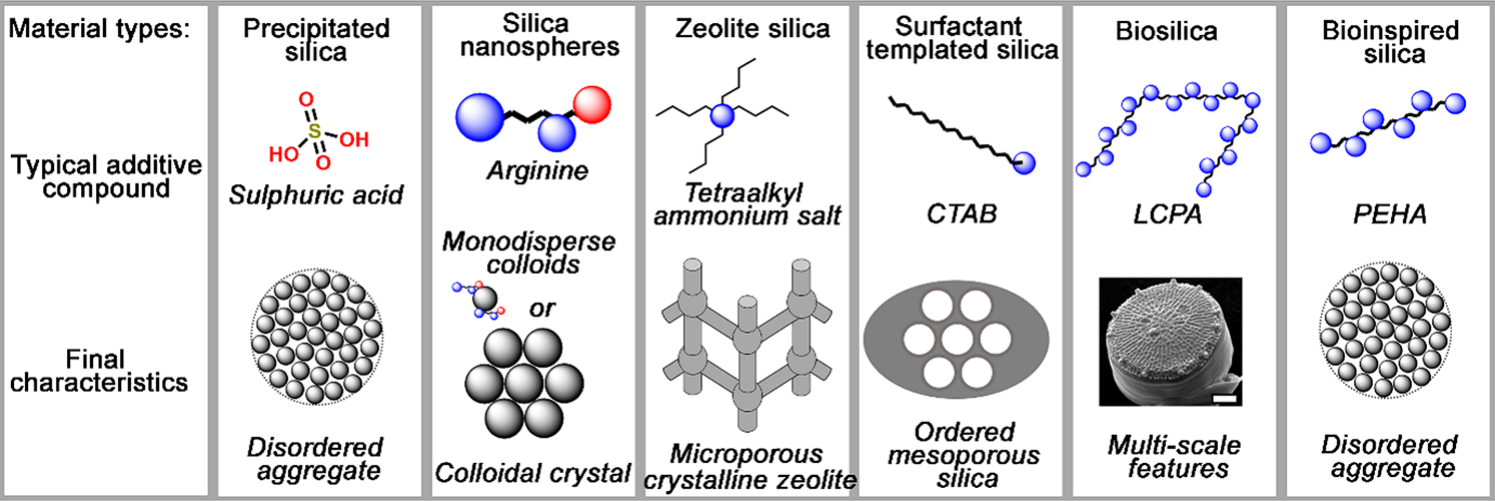
Discrete sol–gel silica product families. Using a variety of additives, most commonly amine-based organic molecules, several families of silica materials developed are shown with controlled particle and pore morphology on multiple length scales. Image reproduced with permission from Manning et al.^28^

Importantly, these broad modes of action are not unique to the BIS system. Recent findings on BIS have contributed to an important associated conceptual step. The starting point, for which, being the recognition that while the design of porous sol–gel silica materials is a thriving research field as depicted in Figure 10, owing to the diversity of properties they can exhibit and potential applications, there has been a lack of joined-up thinking.

In particular, mechanistic insights gained for a particular set of conditions or choice of additive are not translated or generalized across the wider silica family. This matters because, despite the wide range of study into these materials, none can recreate the features and complexity present within naturally occurring biosilica materials and many struggle to offer economically viable or environmentally sustainable routes to scale-up. This lack of a unified approach represented a major barrier to full exploitation of these systems, which relies on the development of a deeper and integrated understanding of the associated formation mechanisms. Subsequent unification of mechanistic knowledge across this expanded porous sol–gel silica family revealed evidence of common driving forces for the structural organization,^28^ which were categorized as

(1) controlling rates of silica precursor hydrolysis and condensation,
(2) forming charge-matched adducts with silicate ions in solution,
(3) self-assembling into mesophases to physically template pores, and
(4) confining the collation of synthesis into specifically shaped vesicles.

**Figure 11:**
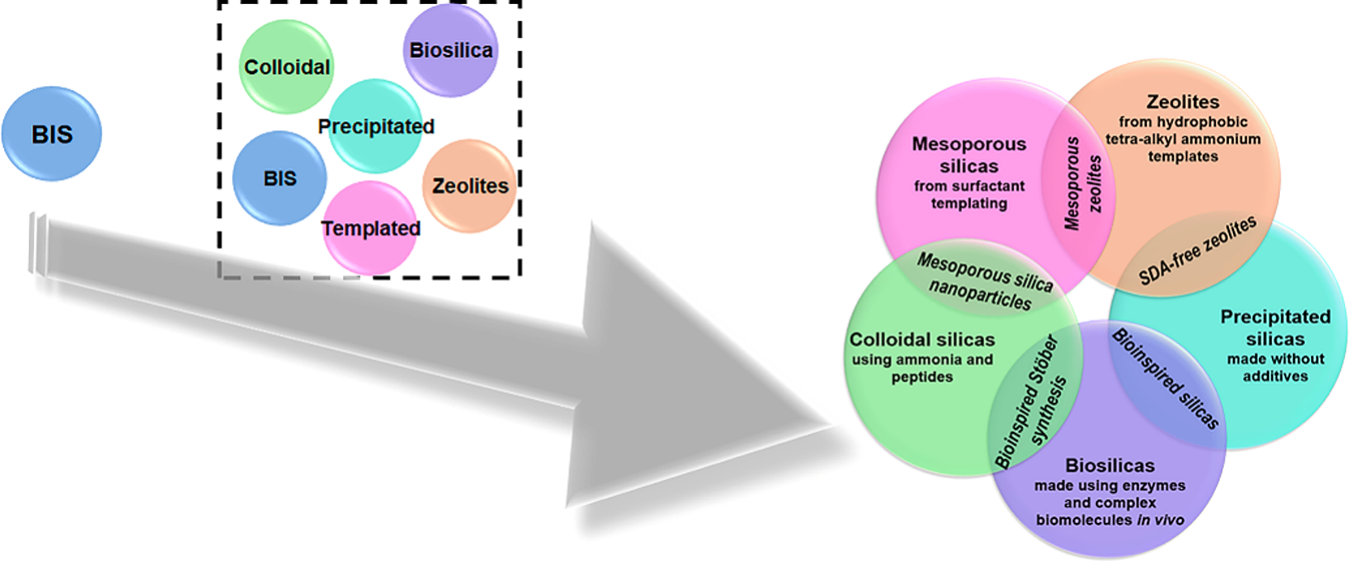
Schematic showing (from left to right) how the knowledge gained from BIS, when applied to discrete sol–gel silica materials, helped unify them into a single sol–gel silica family with clarity on overlaps and intersections. Image in the right is reproduced with permission from Manning et al.^28^

These driving forces enabled the formulation of additive structure–activity relationships for each family and mode. As such, a deeper understanding of BIS has enabled us to complete a map of all types of silicas and helped identify actual and possible hybrid products formed through the combination of available control strategies (Figure 11).

This unification of a wider range of silica materials provides an important framework for the further exploration and exploitation of manufactured silica nanomaterials. The findings are underpinned by robust evidence, and its arguments are presented with practical application in mind. In doing so, the work illuminates the wider relevance and importance of research centered on bioinspired silica, illustrating its potential wider relevance across the entire sol–gel silica system. Further avenues of research will lead to continued increasing understanding of the structure–function relationship between additives and final materials, in turn opening access to increasingly complex and high-value materials.

## Application Fit

### Specialty Silica: Market Complexity

With the progress made on BIS in terms of controlling properties and function, mechanistic understanding, and scale-up, as noted, BIS is maturing into a technology with a potential for commercialization. Understanding this potential for bioinspired silica nanomaterials to compete within the current market profile and indeed to influence its makeup, requires a more detailed view. Development and interrogation of this is its own research challenge. Sources of complexity include

(1) diversity of material properties, grades and costs accessible by varied manufacturing routes,
(2) overlapping material properties and grades, with close equivalents accessible by more than one route,
(3) diversity of applications in terms of critical properties, material function, product sector and specific use,
(4) for a given application, materials may also be in competition with nonsilica chemistries or entirely different technology,
(5) market dynamics, including the possibility of significant new growth, innovation and techno-economic shifts, add further complexity, and
(6) practical influences of commercial interests, intellectual property constraints and investment barriers.

### Market Segmentation and Prospects for Bioinspired Silica Product

While individual applications have specific performance requirements, it is useful in broad terms, to consider the overall market as a series of property–price brackets and, more specifically, porosity–price brackets. For example, lower value markets have lower or less specific porosity requirements than those of higher value. In the current overall market, these brackets may be considered usefully to align approximately with three important manufacturing routes: industrial precipitated silica (IPS), silica gels and sols (SGS), and ordered mesoporous silica (OMS). The third, OMS, being a special subset of SGS.^50^

**Figure 12:**
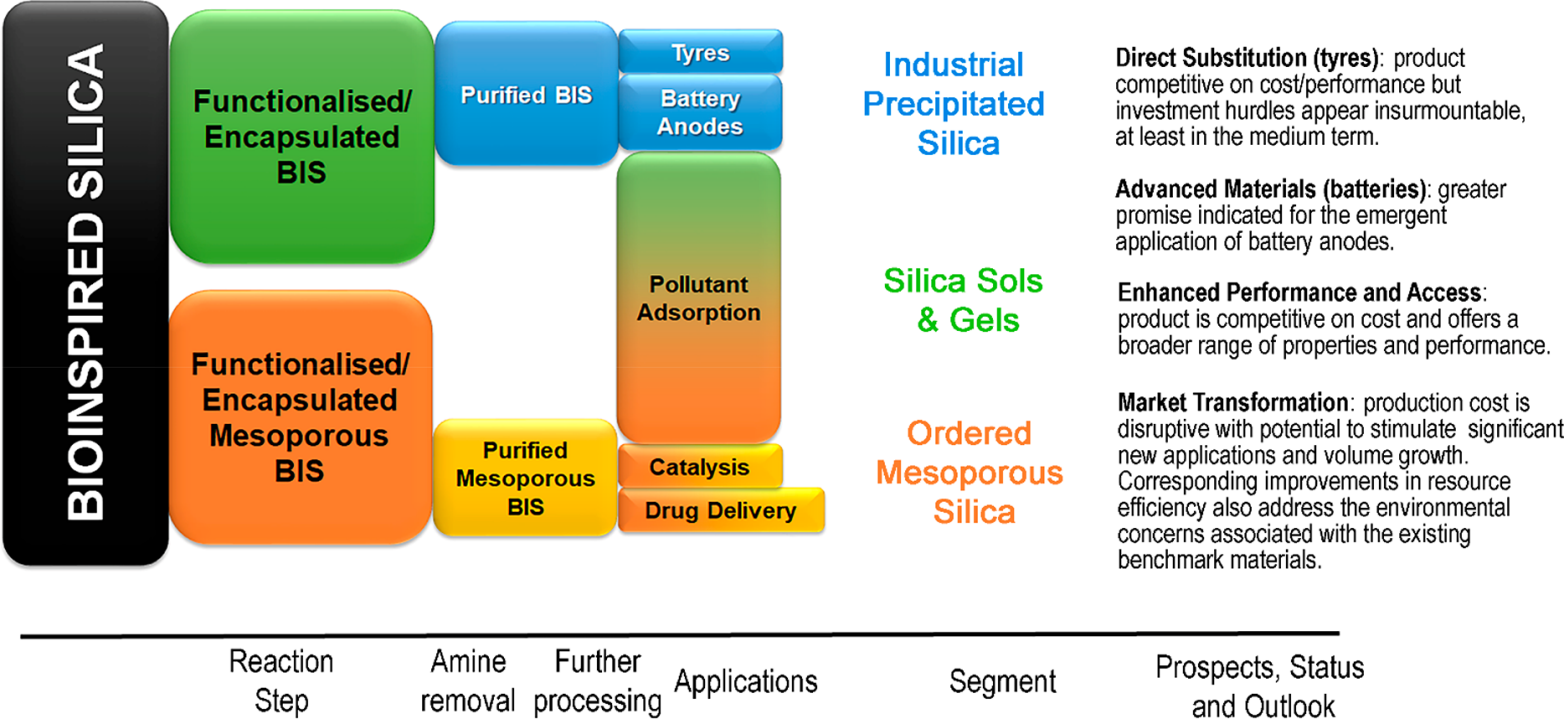
Bioinspired silica nanomaterials: selected material properties, corresponding target applications, and market outlook.

Table 4 and Figure 12 are based on recent studies and highlight how BIS materials can be tailored for these different segments and corresponding market sectors. It considers properties, value, applications, progress toward commercialization, and future opportunities. Key points are as follows

(1) BIS may substitute directly for precipitated silicas in many applications (e.g., tires) and, taking overall production into account, exhibits a similar environmental profile. However, low product value and margins coupled with associated investment and entry-cost mean that significant cost reductions would be required for BIS materials to penetrate these markets.
(2) For many applications where silica sols and gels are currently used, BIS materials offer similar or better cost-performance (again with a similar overall environmental profile). Importantly, they also offer enhanced access and enhanced performance, offering entry to and opening up of new markets. Examples include environmental decontamination, i.e., pollutant removal from water and air.^51−53^
(3) Ordered mesoporous silica is a specialist subset of SGS with applications, for example, in drug delivery. Established laboratory and low-volume benchmarks currently face commercialization barriers due to cost, scalability, and environmental impact. Although analogous BIS materials have not yet been reported, there is potential for novel strategies, utilizing the driving forces discussed earlier, to enable break-through discoveries capable of transforming this nascent market due to a disruptively low-cost, ease of scale-up, and vastly improved environmental performance.

**Table 4:** Market Segmentation and Prospects for Bioinspired Silica Products

Product	Typical properties	Cost and volume	Comparative BIS cost^a^	Prospects	Progress	Outlook
Industrial precipitated silica (IPS)	Surface area 100–300 m^2^/g	Low < £1/kg	Comparable £1.5–2.0/kg	Direct substitution (tires): BIS materials offer comparable cost and performance in traditional applications, the largest of which is in the manufacture of tires.	Early promise in the tire sector, with BIS offering potential for a new supplier to enter an otherwise IP protected market.	Investment hurdles appear insurmountable for traditional applications, at least in the medium term. BIS cost would need to drop further for this calculus to change.
	Pore volume (not generally reviewed)	High Mt/y				
				Advanced materials (batteries): An important and emerging new application is that of Li-ion batteries. BIS materials are an attractive precursor for silicon anodes, showing higher performance than alternative silica materials with comparable cost.^b^	BIS materials have been shown as a viable source for LiB anodes, with the scale-up of Si for anodes demonstrated.	BIS materials show significant promise for emerging application in the manufacturer of LiB anodes; development work continues.
						
Porous sol and gel silicas (SGS)	Surface area 200–600 m^2^/g	Medium £1–10/kg	Comparable or Lower £1.5–2.0/kg	Enhanced access/performance (various): BIS materials offer comparable or reduced cost combined with a broader range of performance options, especially for applications requiring larger pore sizes, or functionalized or encapsulated forms, which do not compromise porosity or increase tortuosity. Therefore, the greatest opportunities lie with improving access to less well established products or to establishing entirely new ones (either within this value bracket or extending it upward).	BIS porous gels have been demonstrated for applications in decontamination of air and water via pollutant adsorption. They have also been tested for hosting catalysts and biocatalysts. They show excellent performance in drug delivery.	Development work continues, focused on specific target markets (environmental) with a view to demonstrating efficacy and value using industry accepted performance testing and protocols.
	Pore volume up to 1.7 cc/g	Medium kt/y				
						
Ordered mesoporous silica (OMS)^c^	Surface area 500–1000 m^2^/g	High > £1000/kg	Disruptively low £5–10/kg	Market transformation (drug delivery): Ordered mesoporous silica is a specialist subset of SGS with applications, for example, in drug delivery.^25,48^ Established laboratory and low-volume benchmarks currently face commercialization barriers due to cost, scalability, and environmental impact.	n/a	BIS has potential to transform this nascent market due to its disruptively low cost, ease of scale-up, and excellent environmental profile. Although, analogous materials have not yet been reported, there is potential for novel strategies, utilizing the BIS driving forces, to direct and enable break-through discovery.
	Pore volume ∼ 0.5–1 cc/g	Low t/y				

a BIS costs are based on data obtained at the lab scale process (unpublished). It is reasonably anticipated that, as for any process, scale-up will reduce costs further.

b Laboratory studies report advanced performance, further investigations of the phenomena and its hypothesized structural explanation are ongoing.^10^

c OMS may be considered as a specialist subset of SGS.

## Summary and Outlook

### Recent Advances

Prior work on biological and bioinspired silica included a large body of biological, biochemical, and discovery work and, alongside this, the conceptual inspiration and growing awareness of the mechanistic complexities pertaining to BIS. The selected advances, covered by this review, build on this platform in the important ways listed (and summarized in Table 5):

(1) first quantitative and systematic investigation of synthesis–product relationships, and associated method development,
(2) extensive chemical and process development, including the main reaction step and an improved mild purification,
(3) first structured investigation of mixing effects, revealing which specific aspects are affected by scale-up,
(4) confirmation of the proposed multistep formation process, which is mediated by charge-matching and found to be supplemented by secondary interactions not previously detected or anticipated,
(5) development and documentation of protocols for preparation of a broad range of functionalized and encapsulated silica materials,
(6) demonstration of scale-up to 40 L leading to provision of larger scale material samples, unlocking new application research, and
(7) continued industrial engagement and market research, leading to identification and investigation of priority sectors and products.

**Table 5:** Summary of Recent Advances Made with BIS, Discussed in This Review, Listed with Corresponding External References

	Advances	Summary	Ref.
1	Demonstrated scalability	Preparation of BIS materials has been demonstrated successfully from 2 mL to 40 L scale. 1–100 g quantities of material can be now made, which unlocked research on silicon/silica anodes and testing for other commercial applications.	(8, 17, 22, 62)
2	Enhanced purification	After the core amine mediated process, the amines may be removed via the new discovery of a mild room temperature acidification to provide pure silica products with associated recovery of the valuable amines for reuse. This invention is important from TEA and LCA perspectives.	(23)
3	Synthesis–product relationships	First steps toward quantitative and systematic insight of BIS reactions and products, including important pH dependencies. Also, methodological development, including a sequential DoE strategy and GSA machine learning.	(16)
4	Mixing effects	A first systematic study of mixing effects revealed that nucleation and aggregation steps are sensitive to meso-mixing.	(33, 38)
5	Functionalized and encapsulated silicas	Prior to purification, amine additives remain incorporated within and on the surface of the BIS nanomaterials, thereby offering a single step route to functionalized silica. With modification to the process (i.e., including metal catalysts, biologicals, drug molecules, etc.), in situ encapsulated silicas may also be formed.	(10, 51−53, 63)
6	Mechanistic studies	A combination of experimental and computational studies established the mediating role of charge-matching between amines and silicates within the formation mechanism with important implications for system understanding, reaction control, and product design.	(28, 45, 46)
7	Characterization studies	Advanced characterization identified significant association between the additives and fully condensed silicon species, leading to higher levels of condensation and greater hydrothermal stability. There are important implications for product performance and mechanistic understanding.	(45)
8	Unified mechanism	A first joined-up mechanistic view of the wider amine-mediated sol–gel silica family. Enables translation of knowledge between the component subtypes and offers the prospect of new, extended, or hybrid materials.	(28)
9	Identification of future challenges	Review paper laying out the sustainability challenges for modern nanomaterials and solutions offered by the bioinspired approach.	(8, 10)
10	Methodology	A reference text for the bioinspired synthesis and sustainable manufacture of inorganic nanomaterials, which includes a widely applicable methodology for discovery to market for green nanomaterials.	(8)

The development approach for discovery to market used for BIS (as shown in Figure 1) was also applied successfully to bioinspired magnetite materials,^54,55^ which in turn illustrates its wider applicability. Related to this was the establishment of a test bed for the rapid prototyping and scale-up of new nanomaterials. Mechanistic insights gained specifically for the BIS system were expanded and integrated to form a unified mechanistic view for the wider sol–gel silica product family, opening channels for academic knowledge transfer between related but previously disconnected subsystems and also identifying common mechanisms and associated structure–activity relations, with potential to drive the development of new and hybrid material types and families. Overall, important strides were made recently in proving the potential of bioinspired silica and in demonstrating how the system can be optimized and tailored. The interactions with industry in defining target specifications and the associated market analysis were crucial to guiding the scientific research.

### Systemic Challenges

The advances listed above provide value of themselves and also, from our systemic view, contribute to the overarching goal of realizing the potential of bioinspired silica. Reflecting on the System-based Product Design (SPD) philosophy, a central goal remains that of establishing a level of detailed (quantitative) mechanistic understanding that enables predictive design, control and scalability of BIS materials and their properties. The broader unified study usefully updated and complemented the earlier conceptual scheme for additive mode of action, in particular, by identifying and highlighting four corresponding driving forces for the structural organization. While for BIS, progress has been made on all four driving forces, a full picture of structural organization is yet to emerge. For example, while the overall (pseudo) chemical kinetics have been reviewed,^56^ specific details of intermediate steps are not measured due to difficulties with currently available analytical techniques. Such knowledge of chemical kinetics will help know exactly:

- which rate steps in the complex pathway are influenced (and which are not),
- which of these steps promote the corresponding aggregation, and
- how exactly this controls templating and determines product properties.

As noted, mesomixing was identified as the dominant mechanism, partly due to the complex pathways of particle formation involving multiple reactions. This means that the established scale-up rules for processes dominated by micromixing cannot be applied for BIS. Hence the correlations between mixing and reaction time scales need to be developed, validated, and used in defining scale-up. Another important gap is that of “reactive assembly.” In self-assembly studies, simulations in particular, participating components are assumed to be nonreactive (chemically “static”). However, in real world systems, silica monomers continue to react, forming a range of oligomers, which change over time. Hence the self-assembly involving these oligomers and additives is dynamic. Understanding this dynamic reactive assembly is crucially important.^57−61^

In summary, the pathway challenge has become increasingly well-defined and understood, but a full picture of structural organization, supported by detailed quantitative kinetics, is yet to emerge. Complexity arises from the need for pathway models to resolve processes of reactive assembly and also to accommodate influence of meso-mixing. It is also important that pursuit of this fundamental knowledge retains sight of the ultimate translational intent. The emphasis here is on gaining “sufficient” and “relevant” mechanistic understanding to enable useful design. These qualifiers hinge on and are directed by the nature and scale of corresponding translational opportunities. This side of the equation must continue to be informed through investigation, collaboration and growing understanding of the markets, applications, performance requirements, and material sustainability and technoeconomics.

Continued systemic challenges are therefore identified as

(1) comprehensive investigation of synthesis–structure and property–performance relationships, and also mixing effects, for BIS materials leading to a complete map of the product design space and supporting development of predictive mechanistic models,^15^
(2) quantify and model reaction pathways and kinetics by studying chemical speciation and its effects on material properties,
(3) fully understanding the techno-economics and life-cycle profiles, and sensitivities across different product types and emerging material grades,
(4) continue working with industry to identify and understand existing and emergent markets and to design and develop suitable materials for specific applications which can be manufactured sustainably, and
(5) develop predictive design capabilities enabling rapid prototyping and scale-up according to specific industry opportunities and requirements.

### Concluding Remarks

**Figure 13:**
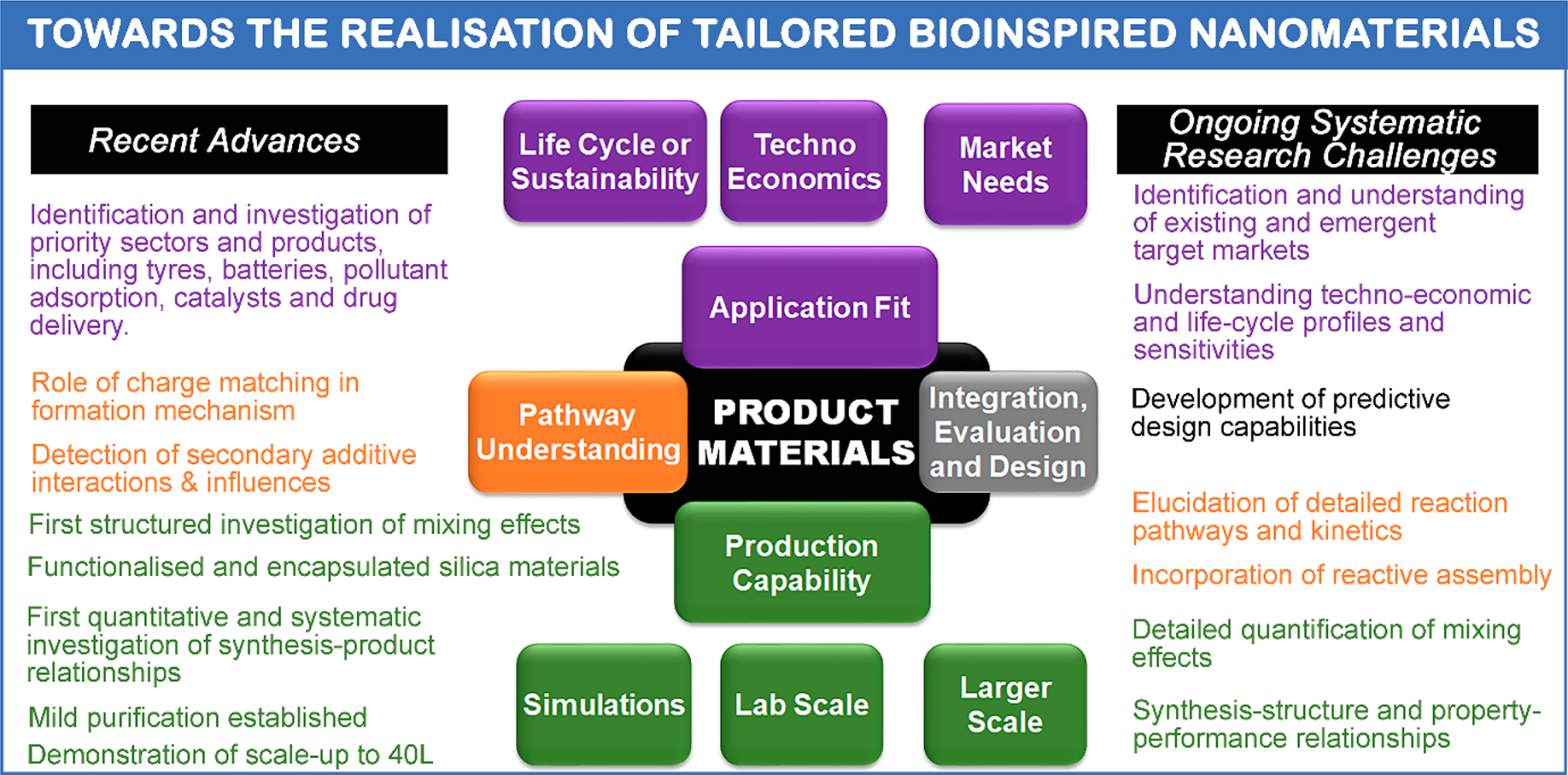
Bioinspired silica nanomaterials: recent advances and systematic research challenges. The text is color coded to match the colors of the boxes.

This paper provides a systemic view of bioinspired silica and a structured view of research to investigate, develop, and exploit the system for the manufacture of silica nanomaterials. It presents important recent advances alongside a supporting contextualisation, which together build on a foundation of many years’ work, and in turn provide a platform for new thinking, knowledge, and materials (Figure 13 and Table 5). It is hoped that this reflection on recent research, the context and associated advances offer insight or inspiration to others. The review has also provided an opportunity for us as researchers to explore different ways of conceptualising this work. There is perhaps a degree of tension throughout between what might be described respectively as “fundamental design” and “translational research” mind-sets. Though closely related, these are not necessarily easy to align. Our experience is that attempting to do so offers creative and formative possibilities.
